# Ca^2+^ release-activated Ca^2+^ (CRAC) current, structure, and function

**DOI:** 10.1007/s00018-012-1072-8

**Published:** 2012-07-17

**Authors:** Martin Muik, Rainer Schindl, Marc Fahrner, Christoph Romanin

**Affiliations:** Institute of Biophysics, University of Linz, Gruberstrasse 40, 4020 Linz, Austria

**Keywords:** STIM1, Orai, STIM1/Orai interaction, Orai gating, STIM1 structure

## Abstract

Store-operated Ca^2+^ entry describes the phenomenon that connects a depletion of internal Ca^2+^ stores to an activation of plasma membrane-located Ca^2+^ selective ion channels. Tremendous progress towards the underlying molecular mechanism came with the discovery of the two respective limiting components, STIM and Orai. STIM1 represents the ER-located Ca^2+^ sensor and transmits the signal of store depletion to the plasma membrane. Here it couples to and activates Orai, the highly Ca^2+^-selective pore-forming subunit of Ca^2+^ release-activated Ca^2+^ channels. In this review, we focus on the molecular steps that these two proteins undergo from store-depletion to their coupling, the activation, and regulation of Ca^2+^ currents.

## Introduction

Among the store-operated channels, the Ca^2+^-release-activated Ca^2+^ (CRAC) channel has been most extensively studied and characterized [65]. In the years 2005 and 2006, the CRAC current-activating and channel-forming units have been identified [16, 40, 76, 97, 107]. Feske et al. [16] have shown that one form of hereditary severe combined immune deficiency (SCID) syndrome is due to a defect in store-operated Ca^2+^ entry. By combining a modified linkage analysis with single-nucleotide polymorphism arrays and a* Drosophila* RNA interference screen, they have identified Orai1 as a CRAC channel-forming protein. Orai1 is part of a family including three homologs. Each of them (Orai1, Orai2, and Orai3) contains a cytosolic N terminus, four transmembrane (TM) segments connected by two extracellular and one intracellular loop, and a cytosolic C terminus. All three Orai proteins form highly Ca^2+^-selective channels within the plasma membrane [74, 77, 97, 105]. Additionally, Orai1, 2, and 3 have distinct properties concerning their inactivation profiles and 2-aminoethyldiphenyl borate (2-APB) sensitivity [12, 42]. One year before the discovery of Orai1, Liou et al. [40] as well as Roos et al. [76], have presented STIM1 as an ER-located Ca^2+^ sensor that is responsible for activating CRAC channels after Ca^2+^ depletion from the ER. They have examined HeLa and* Drosophila* S2 insect cells using an RNA interference-based screen to identify genes that alter thapsigargin-dependent Ca^2+^ entry, which has finally resulted in the identification of two proteins required for Ca^2+^ store depletion-mediated Ca^2+^ influx, STIM1 and STIM2. STIM1, which is the dominant regulator of Orai, contains an N-terminal ER luminal Ca^2+^ binding EF-hand, a single transmembrane domain, and a long cytosolic C-terminal part responsible for interaction with and activation of Orai. The STIM1 homologue, STIM2, possesses approximately 61 % sequence identity with STIM1 with higher divergence at the C-terminal side. At resting state, STIM1 exhibits a tubular distribution within the ER membrane [21, 27]. Upon store depletion, STIM1 oligomerizes and translocates to the cell periphery close to the plasma membrane where it forms punctate clusters and activates Orai/CRAC channels [41, 45, 76, 101]. The communication between STIM1 and Orai has been a highly investigated topic in the past 6 years. In this review, we will focus on the molecular processes and domains of STIM1 and Orai that are required for CRAC current activation, function, and regulation. Regarding the physiological or pathophysiological roles of STIM and Orai that currently emerge, we suggest reading some recent reviews (e.g., [6, 26, 75, 80, 92]).

## STIM1

**Fig. 1:**
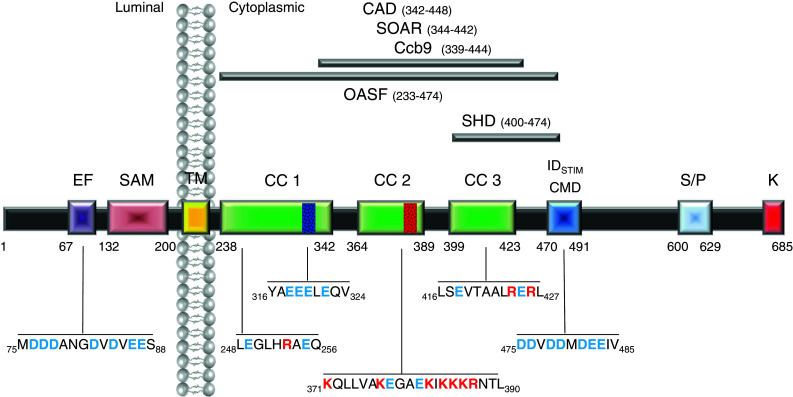
Predicted functional domains within human STIM1. From *left* to *right*: *EF* canonical/hidden EF-hand motif, *SAM* sterile alpha motif, *TM* transmembrane domain, *CC1/CC2/CC3* coiled-coil domains 1–3, *CMD* CRAC modulatory domain, *ID*_*STIM*_ inactivation domain of STIM1, *SHD* STIM1 homomerization domain, *S/P* serine/proline-rich region, *K* polybasic cluster. The minimal functional regions within STIM1 are *highlighted on the top*: *CAD* CRAC activating domain, *SOAR* stim Orai-activating region, *Ccb9* coiled-coil domain region containing region b9, *OASF* Orai-activating small fragment. The respective sequences include amino acids that have been reported to play a crucial role in STIM1 activation and function (charged amino acids are highlighted in *blue* (−) or *red* (+))

The N terminus of STIM1 contains a canonical and a hidden EF hand as well as a sterile-alpha motif (SAM) [90, 91] (Fig. 1). The EF hand, a helix-loop-helix motif with negatively charged residues, binds Ca^2+^ and is therefore able to sense the luminal Ca^2+^ concentration. STIM2 activates CRAC currents upon smaller decreases in the ER Ca^2+^, suggesting this isoform as a feedback modulator that keeps luminal Ca^2+^ in tight limits [5]. Zheng et al. [109] have further examined the EF-SAM domains of STIM1 and STIM2 and concluded that their structural stability difference contributes to the disparate regulation of store-operated Ca^2+^ entry by STIM1 and STIM2. The STIM1 C-terminal part following the transmembrane domain is cytosolic and includes three putative coiled-coil regions, the CRAC modulatory domain CMD [15, 36, 59, 60], a serine/proline- and a lysine-rich region [1, 32, 40, 82]. The C terminus of STIM1 alone is sufficient to interact with and activate Orai1 channels and endogenous CRAC channels [32, 57]. Attempts to elucidate a small STIM1 portion (see Fig. 1) still potent to activate Orai channels have led to the identification of OASF (233–474) [58], CAD (342–448) [66], SOAR (344–442) [106], and Ccb9 (339–444) [34]. These fragments have the second (364–389 CC2) and third (399–423 CC3) coiled-coil domains with additional 39 residues (424–442) in common (Fig. 1). STIM1 fragments including only CC2 and CC3 without the following 39 amino acids are incapable to couple to and activate Orai channels [58]. Analysis of the part downstream CC2 has allowed for the interpretation that the segment 421–474 comprise a cytosolic STIM1 C-terminal homomerization domain (SHD). In the absence of SHD, cytosolic STIM1 fragments preferentially remain in monomeric form [58]. A STIM1 C-terminal deletion mutant (aa 1–440) still forms puncta and co-localizes with Orai1, however, it fails to activate Orai1 currents [66]. Consequently, the residues 421–440 represent the minimal segment maintaining C-terminal STIM1 multimerization.

## Orai

**Fig. 2:**
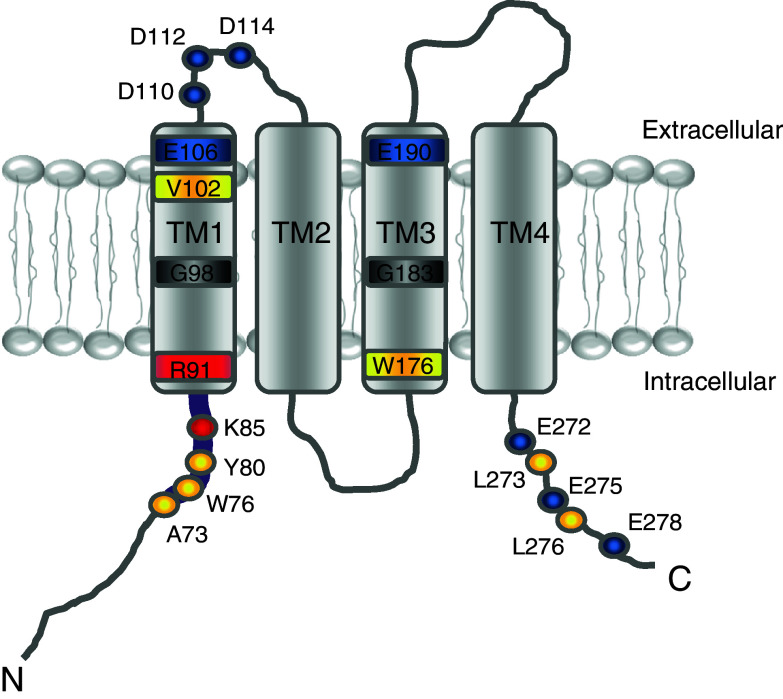
Predicted functional domains within Orai1. Schematic depiction of a single Orai1 subunit with selectively *highlighted* residues that are critical for channel function (*blue* (−), *red* (+), *yellow* (hydrophobic)). In detail, the conserved region (*thick purple line*) on the distal end of the amino terminus contains crucial amino acids for channel gating (K85, R91) as well as Ca^2+^-dependent CaM binding (A73, W76, Y80). Transmembrane domain 1 (TM1) buries the pore-forming residues and the selectivity filter whereas transmembrane domain 3 (TM3) might allosterically affect channel gating. The carboxyl terminus comprises charged (E272, E275, E278) as well as hydrophobic (L273, L276) amino acids that possibly account for coiled-coil domain formation and STIM coupling

Identification of Orai1 (Fig. 2) as the pore-forming subunit of the Ca^2+^ channel allowed for tremendous progress towards the functional and molecular understanding of CRAC. Together with STIM1, Orai1 has been found to fully reconstitute CRAC currents with its known intrinsic biophysical and pharmacological characteristics [110]. The fact that overexpression of the two proteins results in reconstituted CRAC currents has enabled us to address questions about their overall communication [54, 68, 107]. In principle, two pathways are possible as to how STIM transmits the signal of store depletion to the activation of Orai channels in the plasma membrane: either by a simple, direct interaction or via a further molecule [11, 22, 95, 105]. Meanwhile, several independent studies, however, have proven direct binding of soluble STIM1 fragments to Orai, thereby inducing constitutive CRAC currents [32, 34, 57, 58, 66, 106]. In line, biochemical experiments also suggest a direct coupling of the cytosolic domains of the respective proteins [66]. Utilizing purified cytosolic STIM1 fragments, CRAC channel activation has been demonstrated with Orai proteins expressed in isolated vesicles of yeast, and this it has been ruled out that other additional proteins are required in this activation mechanism [110]. Yet, further proteins may have modulatory functions in regulating the STIM/Orai complex. Within the Orai protein, the major binding site for STIM1 is located to a putative C-terminal coiled-coil domain (Fig. 2). Deletions or mutants that interfere with the coiled-coil formation exhibit abolished communication with STIM1 and result in a complete loss of Ca^2+^ entry [3, 18, 38, 57]. In contrast, several N-terminal truncation variants of Orai are still able to co-cluster with STIM1 despite a subsequent loss of channel activation. Thus, it seems that the N terminus of Orai is more likely responsible to gate the channel into the open state rather than providing a dominant binding site for STIM1.

A functional, conducting Orai channel consists of a tetramer, which is either formed by identical subunits or by a combination of distinct Orai isoforms [19, 42, 55, 78]. However, it is still in debate whether Orai1 or Orai3 resides either as non-functional dimers under resting conditions or already exists in a stable tetrameric form. One of the first approaches by Penna et al. [69] has taken advantage of biochemical techniques to propose a dimeric Orai in the resting state. Furthermore, fixation of cells and subsequent single-molecule photobleaching of fluorescently labeled Orai1 has revealed a tetrameric state only in the presence of soluble STIM1 fragments under these conditions. These findings will support a model, where the formation of a functional tetrameric CRAC channel is caused by a linkage of Orai1 dimers that assemble upon binding to STIM1 [69]. In contrast to these findings, a different study by Ji et al. [33] has utilized the same fluorescence-based approach to propose that the formation of a tetrameric CRAC channel occurs independent of STIM1. Here, no obvious changes in the number of photobleaching steps have been observed under conditions where STIM1 is either co-expressed or not [33]. This result is consistent with the observations of Madl et al. who have carried out single-molecule brightness analysis after photo bleaching in unfixed cells to resolve the question of stoichiometry. Laying the focus on the mobile fraction of Orai, they also suggest a tetrameric assembly for Orai1 proteins under resting cell conditions [46]. In this matter, new approaches like the long-awaited crystal structure of Orai may help to finally resolve this question.

## Permeation of Orai and CRAC channels

The Orai channel family exhibits highly Ca^2+^-selective currents and all three isoforms yield almost identical permeation properties [13, 42]. The heterologous expression of each of the three Orai proteins with STIM1 has allowed for comparison of their electrophysiological pore properties with those of endogenous CRAC currents. Ca^2+^-mediated Orai1-3 currents yield a current–voltage relationship with a prominent inward rectification and a high reversal potential of more than +50 mV [54, 74, 77, 105] in line with a similarly positive reversal potential of CRAC currents in RBL mast cells [29]. The Ca^2+^ selectivity of Orai channels is more than 1,000 times higher than the Na^+^ permeation at physiological conditions and the permeation properties recapitulate those of native CRAC currents [13, 29, 42, 96]. In comparison to Ca^2+^-selective currents, conductance for Ba^2+^ is even preferred, but current increase is only transient [70, 93, 103] followed by a declined steady-state current [12, 42]. Orai and CRAC channels exhibit, like other highly Ca^2+^ selective channels, an anomalous mole fraction behavior for Ca^2+^ [29, 68]. This characteristic is assumed to result from a high-affinity binding of Ca^2+^ ions to the selectivity filter at physiological or higher Ca^2+^ levels that prevents monovalent ions from permeation but allows a high rate of Ca^2+^ influx [24]. A drop in extracellular Ca^2+^ will release Ca^2+^ from those binding sites, and the channel becomes readily permeable to monovalent cations. At intermediate Ca^2+^ levels, monovalent permeation is still blocked and Ca^2+^ currents are also very low. Consequently, the conductance of Orai/CRAC channels goes through a minimum when extracellular Ca^2+^ concentrations are increased.

Monovalent cation permeabilities of Orai/CRAC channels include Na^+^ and Li^+^ ions [12, 30, 37, 42]. Na^+^ currents of CRAC, Orai1, and Orai2 channels exhibit an initial maximum peak followed by a subsequent de-potentiation [72]. Orai3 currents instead decline to a much lower extent [12]. A hallmark of Orai/CRAC influx is that the monovalent Cs^+^ ion is almost impermeable, while other Ca^2+^-selective channels, like TRPV6 or L-type Ca^2+^ channels, let Cs^+^ easily pass in a divalent-free solution [25, 98]. This divergence in Cs^+^ permeability results from the difference in pore diameter. L-type Ca^2+^ channels exhibit a diameter of 6 Å [9, 50, 67]; that of TRPV6 is around 5.5 Å [99], however the minimum size of Orai/CRAC pores is much smaller, with 3.9 Å [71, 77, 103]. Thus the Orai/CRAC pore is in the same range as the diameter of Cs^+^ with its hydration shell of 3.8 Å. Hence Cs^+^ ions are apparently small enough to enter the selectivity filter of Orai/CRAC channels, but seem to be sterically hindered. In line with the small pore size, Orai, and CRAC channels yield a very small unitary conductance of 9–24 fS and 6 fS, respectively, in a 2–110 mM Ca^2+^ solution [72, 103, 112].

The initial strategy to discover key residues for Orai Ca^2+^ selectivity has focused on negatively charged amino acids within the first and third TM segment and the first loop of the channel (see Fig. 2). Their acidic side chains may interact with and partially dehydrate Ca^2+^ ions. Even a conservative glutamate-to-aspartate substitution (E106D) within TM1 strongly reduces Ca^2+^ selectivity, but increases monovalent cation permeability [74, 77, 96, 103, 105]. A glutamine or alanine at this position abolishes Orai1/2/3 channel activity and such mutants act in a dominant negative manner [42, 96, 105] and native CRAC currents [22, 74]. Single alanine substitution of one of the negatively charged residues within the first loop or the third TM segment retains the high Ca^2+^ selectivity [74, 77, 96, 105]. Only a glutamate-to-glutamine substitution in TM3 decreases Ca^2+^ selectivity [74, 77, 96], suggesting an allosteric effect on the pore. Within the first extracellular loop segment, a concomitant mutation of all three acidic residues is required to generate a non-selective cation channel [96, 103]. The reduced Ca^2+^ selectivity of these Orai mutants correlates with an increased minimum pore size [103]. Hence, these experiments suggest E106 in the center of the selectivity filter of Orai1 (see Fig. 2), while the other residues may either allosterically interact with, or act in a cooperative manner on, the selectivity filter.

## STIM1/Orai coupling machinery

### STIM1 oligomerization

Total internal reflection fluorescence microscopy visualizes an impressive change in localization and dynamics of fluorescence-labeled STIM1 but also Orai1 proteins, when stores get depleted. At resting cell Ca^2+^ levels, STIM1 is moving fast along the microtubule, whereas a drop of [Ca^2+^]_ER_ dramatically slows down its motility [84]. Store depletion leads to the formation of stable higher-order STIM1 oligomers as suggested by Förster resonance energy transfer (FRET) experiments [41, 47, 57]. This transition displays fast kinetics (*t*_1/2_ ~ 5 s) and involves crucial luminal as well as cytoplasmic STIM1 domains. In detail, the initial trigger for STIM1 oligomerization is Ca^2+^ store depletion where the EF hand loses bound Ca^2+^ (*K*_d_ ~ 200–600 μM) which destabilizes the entire EF-SAM entity [89]. Noteworthy, the STIM1 low-binding affinity for Ca^2+^ perfectly matches with estimated [Ca^2+^]_ER_-dependent activation (*K*_1/2_ ~ 200 μM) which underlines its role as a sensor protein and enables to rapidly respond to store-depletion [45]. The conformational rearrangement of the EF-SAM complex is tightly associated with its homomeric self-assembly [89, 90]. A multimeric state of the luminal domains under low concentrations of Ca^2+^ is in line with a rather high Hill coefficient (~4), which has been calculated for wild-type STIM1 in store-depleted cells [45]. Furthermore, artificial, luminal cross-linking of STIM1 results in its accumulation at the plasma membrane followed by Ca^2+^ influx, thereby demonstrating the causal role of STIM1 oligomerization and Orai channel activity [45]. Taking together, these results support a model in which an initial oligomerization on the luminal site represents an elementary step for subsequent activation of STIM1 [1, 40, 44, 76, 101].

On the cytosolic STIM1 side, a critical role is attributed to internal coiled-coil domains (see Fig. 1) [10]. STIM1 deletion mutants lacking the whole C-terminus are capable of forming dimeric homomers. However, these aggregates appear to be unstable [10]. Addition of the first coiled-coil domain at least supports the formation of stable STIM1 dimers in the resting state although no further oligomerization is visible upon store depletion [10]. Higher-ordered oligomers are only found in STIM1 variants that additionally contain the minimal CRAC activation domain (CAD; 342–448). This is consistent with the finding of a STIM1 homomerization domain (SHD; 400–474) that is required as a functional unit for proper oligomer formation [58]. Hence, this C-terminal multimerization step allows to span the distance between the two adjacent membranes and to facilitate the binding to Orai channels. However, the mechanism as to how the oligomerization of the luminal and cytosolic domains are concerted, requires more detailed studies.

### STIM1 activation and coupling to Orai1

**Fig. 3:**
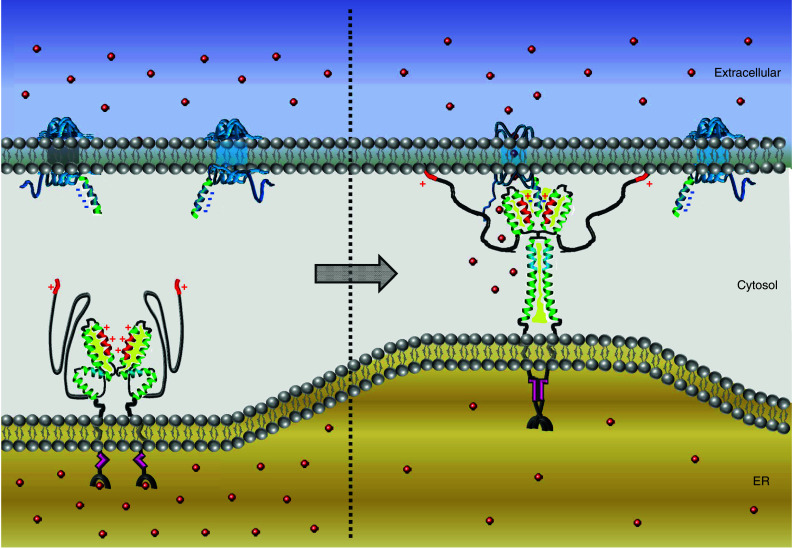
Model for STIM1 activation and coupling to Orai1. This model emphasizes domain interactions mediating the coupling between STIM1 and Orai rather than depicting the full stoichiometry of the complete tetrameric Orai1 assembly. On the left, under resting state conditions in which Ca^2+^-stores are replete, STIM1 is considered to be in a closed conformation. Intramolecular locking involves hydrogen bonds between an inhibitory helix (*blue*) at the C-terminal end of the first coiled-coil with the CAD/SOAR region. The cluster of positive residues (+/*red*) and hydrophobic (*yellow*) interactions via the second and third extended coiled-coil domains arrange the CAD/SOAR dimer in a V-shape form. On the right, Ca^2+^-store-depletion triggers STIM1 oligomerization by refolding of EF-SAM domain followed by a transition of the cytosolic portion into an extended conformation. This causes the rearrangement of STIM1 into punctate clusters and attraction of Orai1 to these regions via a direct interaction of their carboxyl termini. A second additional binding to the N-terminus of Orai1 culminates in channel activation and Ca^2+^ influx

STIM1 aggregation is followed by its translocation from microtubular structures to plasma membrane junctions where it accumulates into discrete puncta [40, 44, 76, 83, 101]. Such puncta formation involves contribution by a lysine-rich cluster at the very end of STIM1 responsible for its attachment to the plasma membrane potentially via binding to phospholipids [41]. This redistribution of STIM1 causes a co-clustering of oppositely located Orai channels that further results in CRAC channel activation. FRET microscopy has revealed close proximity between STIM and Orai at these sites in store-depleted cells [2, 57, 61] and Ca^2+^ imaging together with patch-clamp techniques supplied strong evidence that the resulting Ca^2+^ entry is confined to these restricted areas [45]. These so-called puncta or Ca^2+^ hot spots occurred at sites where the endoplasmic reticulum and the plasma membrane come in close contact. The distance between the two adjacent membranes has been estimated with electron microcopy to be about 10–25 nm [101], whereas the required space for the two key players has been shown to be approximately 4–6 nm for STIM and around 11–14 nm for Orai [96]. Hence, this narrow distance of the membranes allows STIM1 and Orai proteins to directly communicate with each other (see Fig. 3). In the case of Orai, a recent study provides evidence that Orai1 adopts a teardrop-shape form and may be considered as a macro-complex comprised of additional auxiliary units under native conditions [49]. Considering STIM1, the estimated distance is somehow surprising as a simple stretched form of the cytosolic part of STIM1 would require more space to fit within the two membranes. However, two recent independent studies [35, 59] suggest a molecular refolding within STIM1 upon store depletion. As a consequence of STIM1 oligomerization, these authors propose a conformational rearrangement within the cytosolic site of STIM1 (see Fig. 3). Thus, in the closed state, at resting levels, STIM would require much less space in between the two membranes in contrast to the opened unfolded state. In the latter state, it is able to span the larger distance required for binding to the Orai channel at the plasma membrane. The studies by Muik et al. [59] utilize a STIM1-derived conformational FRET sensor that encompasses basically all three coiled-coil domains and the SHD, which has enabled visualizing intramolecular transitions within this cytosolic fragment. In the absence of Orai1, this sensor exhibits a tight conformation, whereas coupling to the channel extends its conformation. Maintaining STIM1 C terminus in a closed conformation involves a complex interplay of crucial hydrophobic [59] as well as charged amino acids [35] within all three coiled-coil domains. More precisely, local disturbances of coiled–coiled segments (L251S, L416S, L423S) or neutralization of charged clusters within these regions (E318/319/320/322A) result in a conformational rearrangement of STIM1 into a more unfolded state leading to constitutive coupling to and activation of Orai1. In contrast, a single point mutation (R426L) that forces STIM1 to remain in a conformationally locked state shows only slight co-localization with Orai1 upon store depletion in line with highly reduced SOCE compared to its wild-type form [59]. Together, these findings suggest a model (Fig. 3) where activation of STIM1 exposes embedded critical binding sites that are shielded by hydrophobic/electrostatic interactions in the closed state [35, 59]. There is strong evidence that this molecular switch might relieve STIM1 second coiled-coil domain enabling its binding to the carboxyl terminus of Orai1 [35]. Mutations of either hydrophobic or charged amino acids in both the C-terminal part of Orai1 as well as the second coiled-coil domain of STIM1 results in an impaired coupling [7, 10, 18, 35, 57, 61]. Thus, the release of CAD/SOAR may provide the basis for a heteromeric coiled-coil/electrostatic interaction, which is the first step and elementary for the overall communication between STIM1 and Orai (Fig. 3). It is of note that, while STIM1 oligomerization, as mentioned in the earlier section, appears to be rather fast, its accumulation at the plasma membrane underlies a certain delay [41, 57]. One simple reason for this observation may be found in the activation process of STIM1. Here, conformational rearrangements on the cytosolic side possibly account for this slower kinetic response.

Interestingly, the extent of CRAC channel activation is not strictly restricted to a defined ratio of STIM1:Orai1 at the junction sites [39]. Recent studies have revealed that a binding of eight STIM1 to a functional channel may allow for maximal Ca^2+^ influx (see Fig. 3). Hence, each Orai subunit, within a tetrameric channel, would couple to a STIM1 dimer, therefore yielding an overall stoichiometry of 2:1 [28, 33, 39].

## 3D structure of STIM1 cytosolic portion

The first crystallographic data of cytosolic regions of STIM1 have just recently been reported [104], representing a further breakthrough towards a molecular understanding of CRAC channel activation. In the following, we will discuss the main findings on the atomic structure of STIM1 in the context with the STIM1/Orai coupling machinery (see also Fig. 3). At the resting state, STIM1 most likely exists as a dimer, based on results obtained from crystallized SOAR protein (344–444) with the interface formed by coiled-coil interactions with the C- and N-termini, respectively, from the other monomer (Fig. 3). In an overall V-shape structure of the SOAR dimer, at the basis of the V structure, C-terminal residues (R429, W430, I433, L436) from one monomer interact with N-terminal amino acids (T354, L351, W359, L347) of the other monomer. The interacting C-terminal residues are consistently located within that region earlier proposed as SHD (400–474) [58]. Mutations within this dimer interface both in SOAR or full-length STIM1 disrupt co-localization with as well as activation of Orai1 [104], though the proposed monomeric state of these mutants has not been directly shown. N-terminal extension of the SOAR domain has led to a longer STIM1 fragment (233–465), which includes the first coiled-coil domain (238–342) suggested to function as an inhibitory region keeping this fragment in a tight conformation [59] and the SOAR domain in an inactive state [35]. Although N-terminal residues (233–306) have not been modeled in the structure, presumably due to a flexible conformation, residues 310–337 represents an α-helical structure [104]. This segment, designated as the inhibitory helix, forms interactions within one STIM1 moiety via several hydrogen bonds between the C-terminal end of the first coiled-coil (322–334) and the N-terminal (343–352) as well as C-terminal (437–440) region of SOAR (see also Fig. 1). These findings differ from the electrostatic model proposed by [35], in which an acidic segment (318–322) of the first coiled-coil domain interacts with a basic segment (382–387) in the second coiled-coil of SOAR to keep STIM1 inactive [35]. Nevertheless, these basic segments are located close to either tip in the overall V-shape structure (see Fig. 3) and are suggested to interact with the negative residues in Orai C-terminus [8, 18]. The structural architecture underlying the reported stoichiometry of 8:4 for STIM1:Orai1 that leads to maximal CRAC current activation [28] is so far elusive, though one may speculate on one Orai C-terminus clamped between the tips of the V-shape SOAR dimeric structure (see Fig. 3). An essential role in Orai gating has also been suggested for its N-terminus [3, 43], yet the molecular mechanism within the STIM1/Orai signaling machinery is not yet clear. Moreover, the hypothesis of Orai N- and C-termini bridged via STIM1 [66] has yet to be demonstrated. It is of note that all three Orai proteins contain a highly conserved N-terminal segment close to the first transmembrane sequence (see Fig. 2). This conserved segment exhibits a region with five basic residues, but it is still open where the respective binding site on STIM1 cytosolic portion is located. It is interesting to note that the electric potential distribution of the SOAR dimer reveals a negatively charged patch close to the base of the V-like structure [104], which might be a candidate for interaction with the basic residues located in the conserved Orai N-terminal segment. Co-crystallization of STIM1 with Orai (fragments) will ultimately help to resolve the molecular interface of the STIM1/Orai coupling.

### Gating of Orai channels

STIM1 binding to the C-terminus of Orai1 is best characterized and likely the dominant coupling site between these two proteins [18, 57]. Yet there is emerging evidence that further cytosolic Orai1 domains are required to gate Orai channels. These include a conserved N-terminal domain starting with residue 74 in Orai1 to the beginning of the first transmembrane segment, and also the second cytosolic loop [3, 86]. To elucidate this N-terminal Orai1-binding site, Park et al. [66] have taken advantage that the CAD/SOAR domain within the C-terminus of STIM1 is more efficient in activating Orai1 currents than the full-length STIM1 C-terminus. The CAD domain has enabled to identify an additional binding to the conserved N-terminal region within Orai1 [66], which is weaker than to the C-terminus. Studies on gating and regulation of Orai3 have revealed a multi-faceted role of the conserved N-terminal region [3]. The latter half of the conserved region (55–66, the analogous region is 80–91 in Orai1) is sufficient for retaining Orai3 activation. A further deletion up to residue 57 abolishes store-operated current activation, while STIM1 binding is only slightly reduced and 2-APB simulation remains preserved [3]. Within the N-terminus of Orai1/3, a single-point mutation close to the first transmembrane segment (K85E in Orai1, see Fig. 2) abolishes store-operated Ca^2+^ currents [43]. The binding between an N-terminal peptide of Orai1 and CAD is only slightly weaker when the N-terminus includes a K85E mutation [43]. Hence, the major role of this lysine consists of transmitting a gating signal to the pore. A second residue in Orai1 affecting gating is arginine 91, localized at the interface between transmembrane (TM) segment 1 and the N-terminus (see Fig. 2). A tryptophan mutation at this position causes the severe combined immunodeficiency (SCID) disease and completely abrogates Orai1 channel activity [16]. Besides R91W, substitution to several other hydrophobic amino acids yields non-functional Orai1 channels [14, 108]. The molecular process(es) as to how these two positively charged residues manage to initiate opening of the gate are still elusive. A possible mechanism may include a conformational switch within the N-terminal segment upon binding to STIM1. Binding of the C-terminus of STIM1 to both the N- and C-terminus of Orai1 may affect the distance of the cytosolic Orai strands and transmit a gating force via residues K85 and R91. Two serines (S89 S90) that are directly located between the two basic amino acids may contribute to the flexibility of the adjacent N-terminal and TM1 segments [14]. Their substitution to more flexible glycines enhances Orai1 currents, while insertion of rigid prolines at this site abolishes these currents [14]. A concerted interplay of the basic residues with the serines may transmit a conformational switch of the N-terminus to the TM1 segment opening the channel.

The TM1 forms the main part of the permeation pathway of the channel, with glutamate 106 in the center of the selectivity filter [52, 111]. Cross-linking experiments of single cysteine mutations in TM1 have demonstrated dimerization for residues at positions 88, 95, 102, and 106. Hence, TM1 is centrally located between Orai1 subunits with an α-helical structure [111]. Residues on the N-terminal half of TM1, located closer to the cytosol, exhibit a weak crosslinking, while the outer part of this transmembrane segment shows crosslinking at selective residues. This suggests that the inner residues have the ability to rotate and thus provide more cross-linking sites, while the outer region (99–104) appears more rigid [111].

The cysteine mutants can also be coupled with methanethiosulfonate (MTS) reagents that introduce upon binding a dramatically increased side-chain. If the MTS bound amino acid contributes to the permeation pathway, channel activity is attenuated. This technique has determined residues in the first loop as well as outside of the selectivity filter (E106) in the ion conducting pathway [52]. However, the narrow pore prevents accessibility to residues more N-terminal to E106 [52]. Alternatively, the small Cd^2+^ ion, with a similar size as Ca^2+^, manages to coordinate cysteine side-chains within TM1. In agreement with crosslinking experiments [111], the Cd^2+^–cysteine binding approach has determined similar residues as well as R91 and G98 for the permeation pathway, suggesting four turns of an α-helical structure of TM1 [52, 108]. The accessibility of Cd^2+^ in contrast to larger MTS reagents is in line with the narrow pore with 3.8 Å in diameter [73, 77, 103].

The more N-terminal residues in TM1 that face the permeation pathway, L95, G98, and V102, are more or less hydrophobic and may contribute to a hydrophobic gate as suggested for other ion channels [56]. Indeed, point mutations of G98 and V102 can result in constitutively active Orai1 currents that no longer require STIM1 for activation [53, 108]. A glycine 98 to aspartate (G98D) or proline mutation yields constitutively active currents. In contrast, an alanine substitution at this position results in non-functional channels [108]. Also, several mildly hydrophobic and polar substitutions for the valine 102 residue result in constitutively active currents. Large hydrophobic amino acids attenuate both the constitutive and STIM1 induced store-operated currents [53]. Hence, manipulation of these residues can lock the channel in a closed or opened conformation and support the model of a hydrophobic gate. However, these spontaneously active channels also exhibit an effect on the selectivity filter revealing reduced Ca^2+^ selectivity due to an increased pore size [53, 108]. While currents derived from constitutive Orai1 mutants at position G98 reverse at 0 mV or even negative potentials, those at residue V102 retain some Ca^2+^ selectivity [53, 108]. An additional substitution of R91W put in the G98D Orai1 mutant results in gain-of-function of the non-functional SCID mutant [108], but lacked channel activity when combined with V102C [53]. In an alternative approach, induced disulfide formation of R91C in a tetrameric Orai channel fails to diminish Ca^2+^ permeation when the pore is extended by the G98D mutant. Therefore, the constitutive G98D mutant likely extends the channel gate wider than that with V102C. These experiments suggest that G98 acts as a gating hinge for channel opening and closing, probably due to its relatively large flexibility on the backbone dihedral angles for conformational changes. This glycine may furthermore allow the rotational mobility of the N-terminal segment of TM1 [111].

The Orai1 V102C mutant revealed an interesting additional aspect: The low Ca^2+^ selectivity of constitutive Orai1 V102C can be enhanced by co-expression of STIM1 [53]. For optimal activation, the tetrameric Orai channel requires eight STIM1 proteins [28, 39]. Tethering of functional STIM1 domains with Orai1-V102C subunits in a 1:1 relation revealed a low Orai Ca^2+^ selectivity. A 2:1 ratio instead yielded typical highly Ca^2+^ selective CRAC currents [53].

Hence, these experiments reveal that STIM1 is able to regulate the ion selectivity and pore architecture of Orai1 channels. Prakriya and coworkers have speculated that the close proximity of the gating residue V102 to the selectivity filter at E106 contributes to the tight coupling of permeation and gating during channel activation [53]. It is of note that all less Ca^2+^-selective mutants are altered in their gating properties as well, and lose the robust fast Ca^2+^-dependent inactivation (CDI) [103]. This fast inactivation process is a hallmark of CRAC channels [17, 30, 113], and will be discussed in more detail in the next chapter. Hence, either the selectivity filter and the gate overlap in the pore structure or mutations within the pore allosterically affect the gating mechanism.

The narrow pore architecture of Orai channels results in a very low single-channel conductance. To optimize Ca^2+^ permeation, the first loop, located extracellular to the selectivity filter (see Fig. 2), creates an increased local Ca^2+^ concentration at the pore entrance [87, 96]. Three negatively charged aspartates within this segment (D110/112/114) attract Ca^2+^ ions and their substitutions to alanines drastically decrease Ca^2+^ selectivity. An asymmetric combination of glutamates and aspartates in an Orai/Orai3 heteromer results in increased Cs^+^ permeation [78]. Hence, at least in an overexpression system Orai1/Orai3 heteromeric channels yield less Ca^2+^ selective channels, however, the physiological relevance awaits further investigations. Single neutralization of the acidic side-chains in the first loop retains the Ca^2+^ selectivity [51, 105]. These experiments suggest that the coordination of the acidic side chains at the pore entrance prevents monovalent outward currents and may also contribute to the high Ca^2+^ selectivity of Orai channels besides E106 [87, 103, 105]. Several cysteine loop mutants form disulfide bonds and dimerize, which suggests a close proximity of two adjacent first loops within an Orai channel. In line, small MTS reagents coupled to the cysteine mutants in the first loop result in decreased currents. Yet, also 6 to >8 Å large MTS probes attenuate currents, which would favor that these residues flank a wide outer vestibule [52]. Hence these results are only in agreement if the first loop is a flexible segment that can undergo conformational changes [52].

A segment that does not line the pore but modulates both selectivity and gating is the third transmembrane helix (TM3) [87]. The tryptophan located close to the cytosolic site of TM3 affects gating and selectivity, as a cysteine substitution at this position (W176C) in TM3 switches the channel in a constitutively active mode concomitant to reducing Ca^2+^ selectivity. A glycine (G183) positioned close to the middle of the TM3 may additionally play a role in modulating the gating. An alanine substitution mutant (G183A) exhibits abolished store-operated activation, but gains 2-APB sensitivity [87]. Although the TM3 does not line the permeation pathway, it might play a role by modulating the gating and selectivity accomplished by TM1.

### Inactivation of CRAC channels

Elevated cytosolic Ca^2+^ provides not only the basis for downstream second messenger processes but might also serve as a pool for internal store replenishment [63]. Store-operated activation of STIM1/Orai1 channels is a fully reversible process [40, 84, 95]. Surprisingly, a simple refilling of endoplasmic Ca^2+^ stores is not sufficient to drive STIM1 into an inactive resting state. There is evidence that STIM1 homomerization and its subcellular distribution is affected by [Ca^2+^]_cyt_ even in an ER-repleted state [47, 81]. In addition to ER store refilling, cytosolic Ca^2+^ elevation is crucial for proper STIM1 de-oligomerization [47, 81]. However, the exact mechanism that transmits changes in [Ca^2+^]_cyt_ to the disassembly of STIM1 oligomers leading to degradation of STIM1 clusters at ER-plasma membrane junction sites is still not completely clear. Notably, although key regulatory cell processes are strongly dependent on the availability of elevated cytosolic Ca^2+^ levels, a cellular Ca^2+^ overload certainly entails the risk of cell death [4]. Accordingly, the existence of an intrinsic negative feedback mechanism termed CDI is indispensable for the limitation of CRAC inward currents [30, 113].

The molecular basis of CDI has emerged to be rather complex, as it involves not only the CRAC channel-forming proteins themselves but also depends on their respective binding stoichiometry. In the over-expression system, a high ratio of STIM1:Orai1 results in normal CDI characteristics, whereas lower STIM1 expression ratios reveal a reduced or even complete loss of CDI [29, 79]. This behavior suggests a concentration-dependent role for STIM1 in this process. One explanation has been provided by the identification of a cytosolic modulatory domain (CMD) of STIM1 (see Fig. 1) that contains a conserved cluster of negatively charged amino acids [15, 36, 60]. Alanine substitution of some or all of the acidic charges in this region diminished CDI [15, 60]. ^45^Ca overlay experiments indicate this domain to form a Ca^2+^-binding pocket of low affinity [60]. The exact molecular mechanism by which CMD contributes to CDI is still unresolved. It is tempting to speculate that CMD is able to interact directly with the CRAC channel [36] or it regulates or shields essential domains within STIM1.

All three Orai homologues exhibit CDI, yet differ significantly in the extent of inactivation. Orai3 currents inactivate most rapidly followed by Orai2 and Orai1 [42]. The subtype-specific diversity of inactivation profiles has helped to identify several segments within the Orai proteins. Towards the extracellular side, alanine mutations of all three acidic residues in the first loop strongly diminish fast inactivation but also decrease Ca^2+^ selectivity [103]. A similar lack of CDI was observed with the Orai1 E106D mutation, which directly targets the selectivity filter. On the molecular level, this effect likely reflects an increase in the pore size of Orai1 and suggests a direct involvement of the selectivity filter in the inactivation process.

On the cytosolic side, the second intracellular loop additionally modulates the fast inactivation process [19, 86]. Substitution of critical amino acids within this region enhances the magnitude of Ca^2+^ influx due to the lack of CDI [86]. Furthermore, a small soluble fragment containing the second loop is able to inhibit Ca^2+^ inward currents in store-depleted cells. Hence, this peptide may either interfere with relevant sites that are needed for proper channel function or can act as a blocking particle occluding the pore from the inside, thereby preventing ion conduction [86]. Transferring the second loop of Orai1 into Orai3 decreases the extent of fast inactivation in this chimera to an Orai1-type CDI [19]. Considering the carboxyl terminus, it seems that three conserved glutamate residues are of significant relevance for channel inactivation [36]. It is of note that all these cytosolic inactivation sites act in a cooperative manner as revealed with various Orai subtype chimeras [19]. Hence, mutations in one domain may also affect the interplay with other regulatory sites.

Interestingly, in contrast to the reconstituted system, native CRAC currents come along with a much more pronounced Ca^2+^-dependent inactivation [15, 113]. This might be either explained by the ability of all Orai subtypes to form heteromeric assemblies, which results in different channel characteristics [19, 78], or with an involvement of further components that contribute to inactivation. Indeed, a crucial role is assigned to calmodulin, which binds in a Ca^2+^-dependent manner to a conserved region within Orai1 N terminus, thereby promoting CDI [3, 19, 60]. Substitutions of single critical amino acids within this segment prevent the coupling of calmodulin to its designated binding site in line with an impaired fast inactivation [60].

### Alternative activation of CRAC channels

Store-operated activation is the most widespread way to stimulate the STIM/Orai channel pathway, however, alternative mechanism are starting to emerge [85].

#### Temperature-dependent STIM1 activation induces Ca^2+^ influx and modulates gene expression

Xiao et al. [102] have shown that STIM1 is activated by changes in temperature, leading to Orai1 mediated Ca^2+^ influx independent of store depletion. Temperatures above 37 °C yield a STIM1 movement into junctions probably due to unfolding of the luminal EF-SAM domain that mimicked store-depleted conditions. Although heating has induced STIM1 clusters, functional coupling to Orai1 is inhibited. The high temperatures may enhance the STIM1 K-rich domain-mediated targeting of STIM1 and at the same time prevent or disrupt CAD domain-Orai1 interaction. Only after cooling below 37 °C, Orai1-mediated Ca^2+^ influx is observed, which therefore represents a temperature-dependent heat-off response of the cell. This heat response is unique to STIM1 and Orai1, and is not only observed in a heterologous expression but also for endogenous CRAC channels of Jurkat T cells. Therefore, it seems that STIM1 serves as a molecular temperature sensor in immune cells.

#### *S*-glutathionylation activates STIM1

In lymphocytes, treatment with oxidants induces an increased intracellular Ca^2+^ level [31]. Hawkins et al. [23] demonstrate that oxidants are able to directly activate STIM1 via* S*-glutathionylation of Cys 56 independent of ER Ca^2+^ levels. This amino acid is located in the luminal part near the EF hand of STIM1. Its* S*-glutathionylation probably reduces the Ca^2+^ binding affinity of the nearby EF hand and consequently leads to STIM1 oligomerization and constitutive Ca^2+^ influx independent of store depletion. The effect of glutathionylation on STIM1 activation indicates that STIM1 is important in redox sensing [85]. Hawkins et al. [23] postulate a mechanism whereby reactive oxygen species (ROS) and Ca^2+^ synergistically modulate inflammatory reactions.

#### Hypoxia-induced acidosis uncouples the STIM/Orai Ca^2+^signaling pathway

Not only hyperoxia affects STIM1 but also oxygen depletion, which leads to hypoxic stress and rapidly and reversibly attenuates STIM1/Orai1-mediated Ca^2+^ influx [48]. The rapid hypoxia-induced decline in cytosolic pH results in an uncoupling of STIM1 and Orai1. This induced acidification directly affects the C-terminal coupling sites of STIM1 and Orai as binding of the SOAR fragment with the channel is decreased [48].

Mancarella et al. [48] propose that acidification opposes electrostatic interactions that mediate STIM1 and Orai C-terminal interaction thereby leading to channel closure. However, under the conditions of hypoxic stress and decreased pH, they do not observe dissociation between STIM1 and Orai1 despite this functional uncoupling. Therefore, Mancarella et al. [48] hypothesize that the interaction between STIM1 and the N-terminal part of Orai1 is still present, while the electrostatic interaction between STIM1 and the C-terminal part of Orai1 is inhibited due to decreased cytosolic pH.

## Conclusions and perspectives

Store-operated Ca^2+^ channels have been investigated in detail over the last two decades, with the molecular key players of the CRAC channels, i.e., STIM1 and Orai1, identified in 2005 and 2006, respectively. This paved the way for thorough characterization of the molecular processes and structure–function relationships central to CRAC current activation, permeation, and inactivation. However, several processes within the STIM1/Orai-coupling machinery are less well understood, urgently awaiting 3D atomic resolution of Orai alone and in complex with STIM1 to provide further insight into their coupling. In particular, the molecular mechanisms as to how STIM1 oligomerization leads to CAD/SOAR exposure, the STIM1 domain(s) interacting with Orai N terminus and the transformation of this interaction into Orai gating are not yet clear.

Another field that has just opened comprises the identification of further proteins modulating or participating in the STIM1/Orai signalplex. Recently, SARAF [62], junctate [88], and Surf4 [20] have been identified that modulate STIM1 oligomerization and clustering. The Ca^2+^-activated adenylate cyclase (AC8) has been identified as an integral component of the STIM1/Orai1 signalplex [100] further underscoring the concept of a Ca^2+^-signaling microdomain [64].

Another topic related to STIM/Orai covers their physiological as well as pathophysiological role in various tissues and clearly shows an exponential rise in publications. Related to this, the development of drugs specific to the STIM/Orai system [94] though scarce at the moment, is expected to increase particularly with their emerging role they play in physiological as well as pathophysiological processes.
